# Efficacy of Siddha Therapeutics on Mantha Sanni (Autism Spectrum Disorder) Among Pediatric Patients: An Interventional Non-randomized Open-Label Clinical Trial

**DOI:** 10.7759/cureus.47128

**Published:** 2023-10-16

**Authors:** Preetheekha Elangovan, Gomathi Ramasamy, Meenakshi Sundaram, Meenakumari Ramasamy

**Affiliations:** 1 Department of Paediatrics, Jain's Clinic, Chennai, IND; 2 Department of General Medicine, National Institute of Siddha, Chennai, IND; 3 Department of Paediatrics, National Institute of Siddha, Chennai, IND; 4 Department of Pharmacology, National Institute of Siddha, Chennai, IND

**Keywords:** microbiota, yegamooli thylam, siddha, mantha sanni, autism spectrum disorder (asd), amukkara chooranam

## Abstract

Background: Autism Spectrum Disorder (ASD) refers to a collection of neurodevelopmental disorders that affect brain development and can lead to various psychological imbalances in caregivers of affected children. Siddha formulations have been shown to have a role beyond the physical body and play a significant role in managing *Mantha sanni*or ASD. The objective of this study was to examine the impacts of *Amukkara chooranam and Yegamooli thylam* in the pediatric population diagnosed with ASD.

Methods: This was a prospective, interventional, non-randomized, open clinical trial involving 30 patients who met the inclusion and exclusion criteria. Patients received *Amukkara chooranam* at a dose of 300 mg for ages 3-4 years, 500 mg for ages 5-7 years, and 1 gm for ages 8-12 years, twice a day with honey for 90 days, and *Yegamooli thylam* was administered using the Thuvalai external manipulation technique once a day for 90 days. Scoring by the Indian Scale for Assessment of Autism (ISAA) was documented at the end of the 0th day, 45th day, and 90th day.

Results: The scores were compared at each follow-up, and a statistically significant difference was found at the end of the 90th day of treatment with *Amukkara chooranam* and *Yegamooli thylam* (P < 0.05). The statistical analysis included calculating the mean and standard deviation of the clinical assessment, parameters both before and after the treatment were 37.66667 ±13.82485.

Conclusion: The treatment with *Amukkara chooranam* and *Yegamooli thylam* resulted in a clinically significant improvement in clinical assessment parameters in children with ASD

## Introduction

Autism Spectrum Disorder (ASD) is a multifaceted developmental condition characterized by enduring difficulties in social communication, limited areas of interest, and repetitive behavioral patterns [1]. In 2018, a comprehensive study conducted at 11 different Autism and Developmental Disabilities Monitoring (ADDM) sites in the United States (Arizona, Arkansas, California, Georgia, Maryland, Minnesota, Missouri, New Jersey, Tennessee, Utah, and Wisconsin) revealed varying rates of ASD prevalence among children aged eight years. The prevalence ranged from 16.5 cases per 1,000 children in Missouri to 38.9 cases per 1,000 children in California. The collective ASD prevalence across all sites was calculated at 23.0 cases per 1,000 children, equivalent to one in every 44 children. Furthermore, it was observed that ASD was notably more prevalent among boys, with a rate 4.2 times higher compared to girls [2].

The Center for Disease Control and Prevention (CDC) recommends the use of various medications, including Alpha2-adrenergic agonists, opioid antagonists, psychostimulants, and serotonin reuptake inhibitors, for the treatment of ASD in children. However, it should be noted that the effectiveness and safety of these medications in children with ASD are not as extensively studied. Furthermore, these medications may have limited efficacy and a higher likelihood of causing adverse effects in children with ASD [3]. At present, the utilization of Complementary and Alternative Medical (CAM) treatments is common for children diagnosed with ASD [4]. However, it is essential that these treatments are supported by evidence-based research to ensure their effectiveness and safety. According to Siddha literature, the clinical characteristics of ASD can be likened to the concept of "*Mantha sanni*" [5].

*Mantha sanni* is a kind of delirium in children caused by fever due to indigestion [6]. Mounting evidence supports the existence of a connection between ASD and the gut-brain axis. The gut-brain axis denotes the intricate communication network involving the gut, brain, nervous system, hormones, and immune system. Research has indicated that individuals with ASD frequently experience gastrointestinal problems, including inflammation and disturbances in gut microbiota, which can impact the functioning of the gut-brain axis [7].

The Siddha medical tradition encompasses a renowned polyherbal formulation termed *Amukkara chooranam*, featuring a complex amalgamation of seven botanical constituents, extensively employed within the Siddha system for an extended span of time. Predominantly enriched with Amukkara (*Withania somnifera*), commonly recognized as ashwagandha, this composition exhibits noteworthy therapeutic efficacy in ameliorating a diverse array of afflictions including anxiety, depression, gastrointestinal maladies, as well as impairments pertaining to both reproductive and neuroendocrinological domains. The enduring utilization of this formulation within Siddha medicine substantiates its remarkable proficiency in addressing multifarious health conditions [8]. Research findings have indicated that *Withania somnifera* may exert a favorable influence on gut microbiota. These studies propose that *Withania somnifera* has the potential to enhance the population of beneficial bacteria, including *Lactobacillus* and *Bifidobacterium*, while concurrently reducing the presence of detrimental bacteria such as *Escherichia coli*. Additionally, *Withania somnifera* may help to reduce inflammation in the gut, which can contribute to overall gut health [9].

In order to establish empirical evidence regarding the clinical efficacy of Siddha herbal medications, it becomes imperative to undertake clinical trials to document the effects of Siddha formulations on children diagnosed with ASD. The study aimed to investigate the impact of traditional Siddha formulations *Amukkara chooranam* and *Yegamooli thylam* on the clinical condition of children with ASD, with a focus on evaluating changes in their symptoms over a 90-day intervention period.

## Materials and methods

Study design and settings

A prospective, interventional, nonrandomized, and open clinical trial was carried out at the Ayothidoss Pandithar Hospital, which is affiliated with the National Institute of Siddha, Chennai, Tamilnadu, India. The trial protocol received official approval from the Institutional Ethics Committee (IEC) of the National Institute of Siddha under the grant number NIS/IEC/14/2018-19/13-20.09.18. The trial was carried out in adherence to the principles set forth in the Declaration of Helsinki. Prior to the initiation of the study, the caregivers of the participants were required to provide written informed consent. The participants had the autonomy to voluntarily withdraw from the study at any point, without any impact on their ongoing healthcare provider relationship. The trial was duly registered in the Clinical Trial Registry of India under the identification number CTRI/2019/12/022286.

Trial participants

Inclusion criteria for the participants were as follows: the study participants consisted of children between the ages of three and 12 years who had received a clinical diagnosis of ASD and met diagnostic symptoms of poor eye contact, inappropriate emotional response, acquired speech or lost it, engage in stereotyped and repetitive behavior, and unusually sensitive to sensory stimuli at the time of inclusion into the study. Exclusion criteria were as follows: co-occurring conditions such as attention deficit hyperactivity disorder (ADHD), seizures and epilepsy, cerebral palsy, as well as hearing and visual impairments. Individuals who had congenital anomalies or were diagnosed with other severe illnesses were also excluded from the study population. Withdrawal criteria included instances such as intolerance to the administered drug and the emergence of adverse reactions throughout the course of the trial, poor patient compliance or instances of patients not adhering to the treatment plan, and cases where patients expressed unwillingness to continue participating in the clinical trial.

Interventions

**Figure 1:**
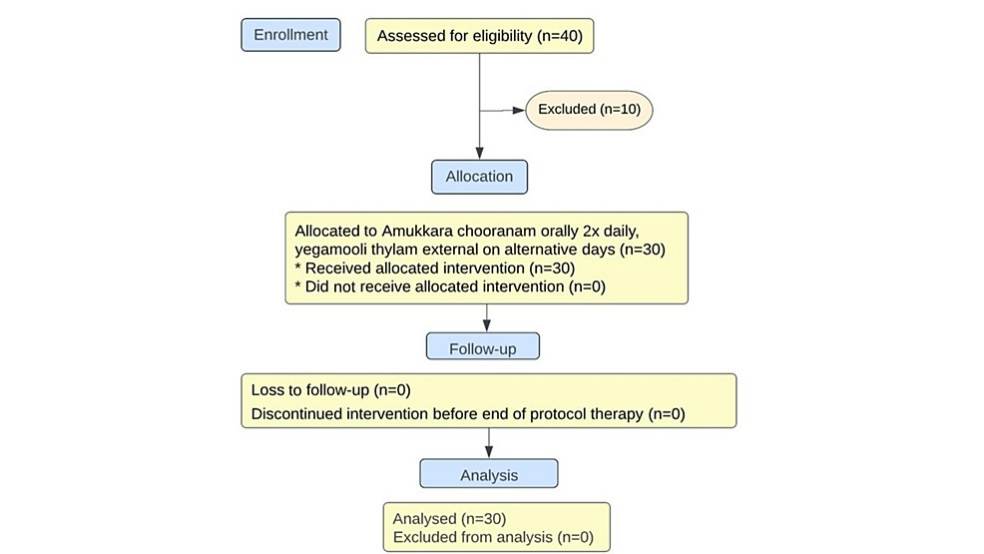
Consort flow chart

Thirty children received *Amukkara chooranam* (Figure 1) [10] at a dose of 300 mg for ages 3-4 years, 500 mg for ages 5-7 years, and 1 gm for ages 8-12 years, twice a day with honey for 90 days and *Yegamooli thylam* [11] as Oleation therapy (Thuvalai) was given to 30 children daily in the external therapy room of National Institute of Siddha (NIS) for 90 days. Oleation therapy entails the systematic application of specially formulated medicated oil to the entirety of the body, serving as an intricate therapeutic modality [12]. Patient adherence was diligently documented on a daily basis using the Drug Compliance Form. Clinical parameters were recorded at specific intervals, namely the 0th day, 45th day, and 90th day, employing the scoring system provided by the Indian Scale for Assessment of Autism (ISAA) [13]. The occurrence of any adverse effects resulting from the medications was vigilantly monitored and meticulously documented within the patient folder, utilizing an adverse effects form.

The drugs utilized in the study were procured from a reputable country shop located at Broadway in Chennai, Tamilnadu, India, and the authenticity of the raw drugs was verified by the Medical Botanist of the National Institute of Siddha. The medications were prepared in the Gunapadam laboratory at the National Institute of Siddha. To prepare *Amukkara chooranam*, the raw drugs undergo a purification process [14], followed by individual grinding and sieving through a fine cloth. Subsequently, the powdered components are thoroughly blended with cane sugar to form the final composition. In the preparation of *Yegamooli thylam*, Sivanaarvembu samoolam (*Indigofera aspalathoides*) is first powdered. The purified Velluli (*Allium sativum*), Vasambu (*Acorus calamus*), and Sivanaarvembu (*Indigofera aspalathoides*) powders are then combined with Gingelly oil. This mixture undergoes a boiling process and is gradually reduced to mezhugu patham. The final medicines are stored in glass containers that have been authenticated by the respective guide to ensure their integrity and completeness (Table 1).

**Table 1:** Composition and ingredients of Amukkara chooranam and Yegamooli thylam administered for ASD children ASD: Autism Spectrum Disorder

Composition of *Amukkara Chooranam*		
Vernacular/Tamilname	Botanical Name	Quantity
Nattu sarkarai	Cane sugar	1280 grams
NattuAmukkara	Withania somnifera	640 grams
Sukku	Zingiber officinale	320 grams
Thippili	Piper longum	160 grams
Milagu	Piper nigrum	80 grams
Elam	Elateria cardamomum	40 grams
Sirunaga poo	Mesua nagassarium	20 grams
Lavangam	Syzygium aromaticum	10 grams
Composition of *Yegamooli thylam*		
Sivanaarvembu	Indigofera aspalathoides	325 grams
Velluli	Allium sativum	35 grams
Vasambu	Acorus calamus	35 grams
Nallenai	Gingelly oil	1.3 litres

Sample size

A total of 40 patients with ASD were screened, out of the eligible participants, a total of 30 subjects who met the inclusion criteria were recruited for the study.

Examination

The acquisition of demographic information was carried out through a questionnaire administered to the caregivers of the children. Anthropometric parameters such as height, weight, and mid-arm circumference were measured. With respect to habits, bowel movements were recorded prior to the initiation of the study. Clinical parameters were assessed through observations, interviews with the caregiver, and testing of the child, which lasted for a duration of 20-30 minutes.

Statistical analysis

The data is presented as Mean ± SD. To assess the impact of *Amukkara chooranam* and *Yegamooli thylam* from the initial assessment to the follow-up, statistical tests including paired t-tests and repeated measures of ANOVA were employed. The predetermined level of significance for the study was set at P < 0.05. Statistical analysis was conducted using GraphPad Prism software (Version 6.01) (GraphPad Software Inc., San Diego, CA).

## Results

Baseline profile of the study participants

Data from a total of 30 subjects was recorded. The majority of subjects were male children (n 26; 87%). The mean and standard deviation (SD) of height (cm) was 114.43 ± 16.26, weight (kg) was 20.33 ± 8.14, and mid-arm circumference (cm) was 14.76 ± 4.58. Age distribution was as follows: three to six years (n 20; 67%), seven to nine years (n 4; 13%), and 10-12 years (n 6; 20%). In developmental milestones, gross motor was found to be normal whereas fine motor, speech, social, and emotional milestones were delayed in all subjects (n 30; 100%). In habits, irregular bowel movements were reported in all subjects (n 30; 100%) (Table 2).

**Table 2:** Baseline profile of study participants

Parameters	Indicator		Values
			Total N=30
Gender	Male		26(87%)
	Female		4(13%)
Age distribution	3-6 years		20(67%)
	7-9 years		4(13%)
	9-12 years		6(20%)
Height (cm)			114.43 ± 16.26
Weight (kg)			20.33 ± 8.14
Mid-arm circumference (cm)			14.76 ± 4.58
Developmental milestones	Gross Motor	Delayed	0
		Normal	30(100%)
	Fine Motor	Delayed	30(100%)
		Normal	0
	Speech	Delayed	30(100%)
		Normal	0
	Social and Emotional	Delayed	30(100%)
		Normal	0
Habits	Irregular Bowel movement		30(100%)

Clinical outcomes and follow-up

**Figure 2:**
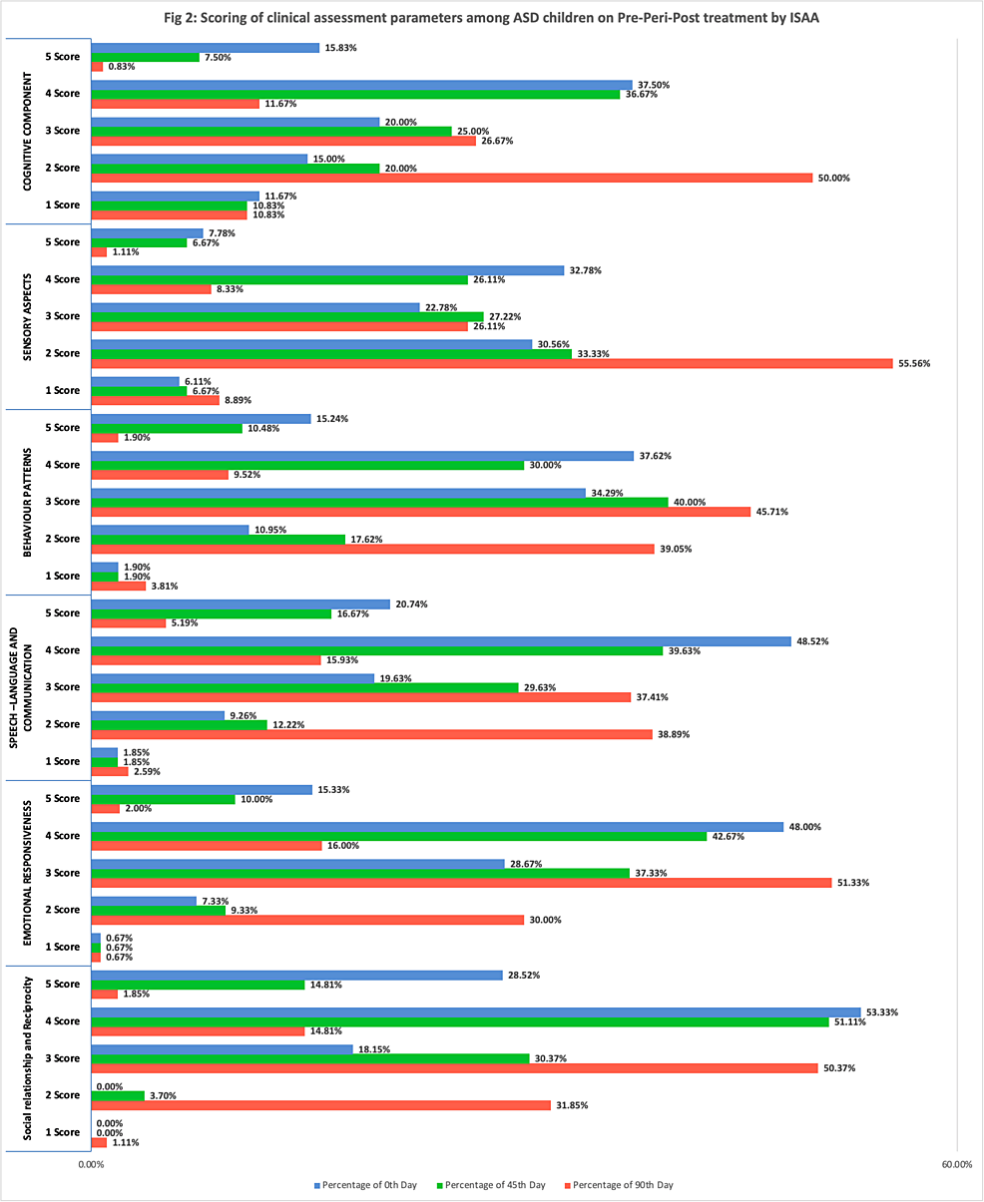
Scoring of clinical assessment parameters among ASD children on pre-peri-post treatment by ISAA ASD: Autism Spectrum Disorder; ISAA: Indian Scale for Assessment of Autism

The clinical parameters were systematically documented on the 0th, 45th, and 90th days, showcasing the progression of changes over time. Notably, significant improvements were observed across all domains of the Indian Scale for Assessment of Autism (ISAA) from the initial assessment to the 90th day. These improvements included a 31% enhancement in social relationship and reciprocity, a 29% advancement in emotional responsiveness, a 25% amelioration in speech language and communication, a 25% enhancement in behavioral aspects, a 27% improvement in cognitive impairment, and a 22% enhancement in sensory aspects. There was a standard improvement from baseline to follow-up (Figure 2).

A meticulous comparison between the initial symptoms and the state at the conclusion of the 90-day treatment period of *Amukkara chooranam* and *Yegamooli thylam* administration revealed a statistically significant difference (P < 0.05). In the context of repeated measures ANOVA, a comparison between the 0th day and the 45th day revealed a mean difference of 8.967. The standard error was calculated as 1.018, with a significance level of 0.000. The 95% confidence interval for the difference ranged from 6.885 to 11.048. Similarly, when comparing the 0th day with the 90th day, a mean difference of 37.667 was observed. The standard error was 2.524, and the significance level was 0.000. The 95% confidence interval for the difference was found to be between 32.504 and 42.829.

**Figure 3:**
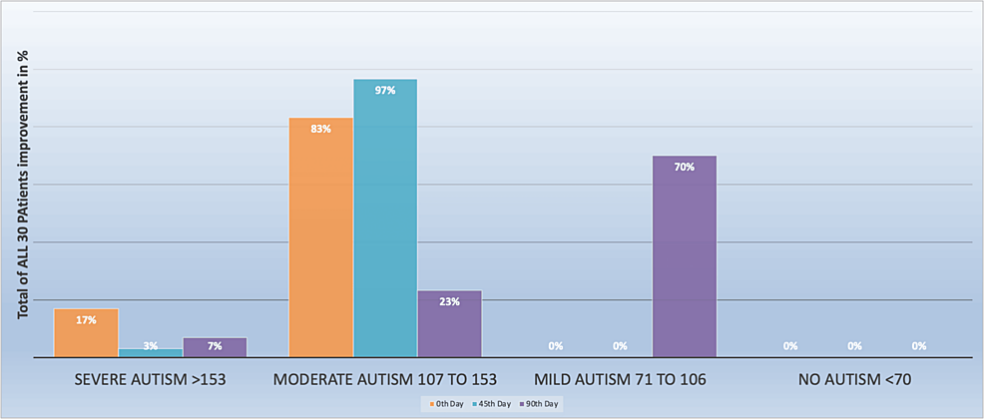
Total percentage improvement in patients with different autism severity levels

**Figure 4:**
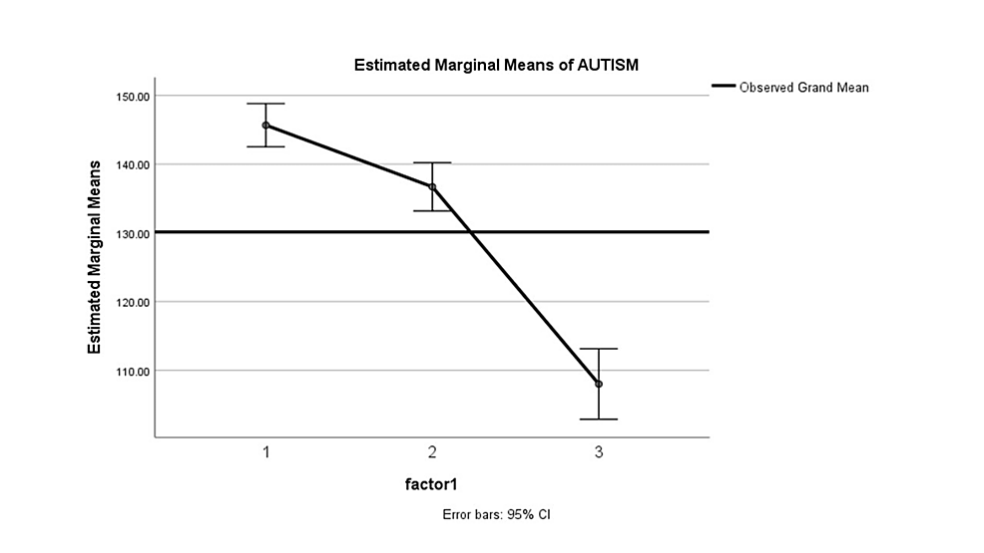
Estimated marginal means of Autism

Furthermore, in the paired sample test, the mean and standard deviation of the assessed parameter before and after treatment were calculated to be 37.66667 ± 13.82485. The standard error of the mean was determined to be 2.52406, and the 95% confidence interval for the difference ranged from 32.50438 to 42.82895. The t-value obtained was 14.923, with a degree of freedom of 29. The significance level (2-tailed) was determined to be 0.000. It is shown in the (Table 3, Figures 3-4).

**Table 3:** Statistical analysis

Pairwise Comparisons (Repeated measures ANOVA	Mean Difference (I-J)	Std. Error	Sig.^b^	95% Confidence Interval for Difference^b^				
Measure:				Lower Bound	Upper Bound			
Initial vs 45th day	8.967^*^	1.018	0	6.885	11.048			
Initial vs 90th day	37.667^*^	2.524	0	32.504	42.829			
Paired Samples Test	Mean	Std. Deviation	Std. Error Mean	95% Confidence Interval of the Difference		t	df	Sig. (2-tailed)
				Lower	Upper			
Initial vs 90th Day	37.66667	13.82485	2.52406	32.50438	42.82895	14.923	29	0

Adverse drug reactions

No significant adverse reactions were observed, and there were no instances of subject withdrawal during the trial.

## Discussion

The field of Siddha medicine has gained acknowledgment and endorsement from esteemed organizations such as the World Health Organization (WHO) and the Indian Government. It has witnessed a notable surge in research endeavors dedicated to exploring its efficacy and safety, with a specific focus on its potential in the management of ASD among pediatric patients [15,16]. Clinical assessments using the Indian Scale for Assessment of Autism (ISAA) have demonstrated promising results in all domains of ASD symptoms with the use of Siddha formulations *Amukkara chooranam* and *Yegamooli thylam* among paediatric patients.

Social relationship and reciprocity

The percentage distribution of scores shows an improvement in this domain over time. For instance, the percentage of participants with a score of 3 increased from 18.15% on the 0th day to 30.37% on the 45th day and further to 50.37% on the 90th day, indicating an improvement in social relationship and reciprocity over the course of the study.

Emotional responsiveness

In this domain, there is a noticeable improvement as well. For example, the percentage of participants with a score of 4 increased from 16.00% on the 0th day to 42.67% on the 45th day and 48.00% on the 90th day, suggesting an enhanced level of emotional responsiveness throughout the study.

Speech, language and communication

Improvement is observed in speech, language, and communication skills. For instance, the percentage of participants with a score of 3 increased from 19.63% on the 0th day to 29.63% on the 45th day and further to 37.41% on the 90th day, indicating enhanced communication abilities over time.

Behavioural patterns

This domain also shows improvement. The percentage of participants with a score of 3 increased from 34.29% on the 0th day to 40.00% on the 45th day and 45.71% on the 90th day, signifying improved behaviour patterns during the study.

Sensory aspects

Improvement is evident in sensory aspects. For instance, the percentage of participants with a score of 2 increased from 22.78% on the 0th day to 27.22% on the 45th day and 26.11% on the 90th day, indicating an improved sensory experience over time.

Cognitive component

Improvement is observed in cognitive abilities as well. For example, the percentage of participants with a score of 2 increased from 15.00% on the 0th day to 20.00% on the 45th day and 26.67% on the 90th day, suggesting enhanced cognitive functioning during the study. It is shown in Figure 2.

The children with moderate autism showed the highest improvement of 97% by the 45th Day, while those with severe autism showed the least improvement of 3%. The 90th Day data suggests that 70% of patients with mild autism improved significantly, while patients with moderate autism had a decrease in improvement compared to the 45th Day, while the patients with moderate autism had a decrease in improvement by 7% compared to 45th day as shown in Figure 3. Figure 4 visually represents how the estimated marginal means for each factor (Factor 1, Factor 2, and Factor 3) change over time (0th day, 45th day, and 90th day) while accounting for the influence of other factors. It allows you to see trends and differences in outcomes for each factor on these specific days. The efficacy of the trial medicine demonstrated statistical significance (P < 0.05) and is consistent with findings from similar studies [17]. 

Previous research studies have identified the beneficial effects of *Withania somnifera*, which is a key ingredient found in *Amukkara chooranam*. These studies have demonstrated its potential in safeguarding motor- and cognition-related behaviors, as well as reducing oxidative stress markers in rodent neuropathology models. Studies have identified the beneficial effects of *Withania somnifera* (a key ingredient of Amukkara chooranam) in protecting motor- and cognition-related behaviours, as well as brain oxidative stress markers in rodent neuropathology [18]. Additionally, the microbiota-gut-brain axis has been found to play a bidirectional role in ASD susceptibility, with genes involved in synaptic transmission and immune pathways affecting the microbiota [19]. The ingredients present in *Amukkara chooranam* have been found to have positive effects on the gut microbiota, including promoting the growth of beneficial bacteria and promoting a healthy balance of the gut microbiome, have been found to have anti-inflammatory actions [18,20-24]. The application of Oleation therapy, as utilized in Siddha medicine, aligns with other studies that suggest its potential for purifying and rejuvenating the body, alleviating anxiety, minimizing adverse effects on the central nervous system, and mitigating toxins and mental fatigue [25].

Additionally, an acute toxicity study conducted on *Amukkara Chooranam* revealed its non-toxic nature, as it demonstrated no adverse effects when administered orally to mice at doses up to 2000 mg/kg and neither acute nor subacute oral administration caused significant changes in gross behavioural effects in rodents [26]. In the current study, none of the subjects reported any adverse events during the trial and there was no withdrawal. Overall, Siddha medicine has a significant role in managing neurobehavioural symptoms in ASD, reducing inflammation in the gut, and contributing to overall gut health.

## Conclusions

To sum up, the treatment using *Amukkara chooranam* and *Yegamooli thylam* has demonstrated a clinically significant improvement in clinical assessment parameters from the baseline (p < 0.05). One of the limitations of this study is the small sample size, involving only 30 patients. While we acknowledge that the study's small sample size, comprising only 30 patients, is a primary limitation, we view this preliminary data as a foundation upon which to design and conduct larger-scale trials in the future. The Siddha system of medicine provides healthcare support for promoting the well-being of ASD children, which can lead to an improvement in their overall quality of life.
